# Sigmoid intussusception and intestinal obstruction secondary to lipoma in an elderly woman: A case report

**DOI:** 10.1097/MD.0000000000046212

**Published:** 2025-12-12

**Authors:** Chenchen Zhang, Dong Zhai, Xiaojuan Tong

**Affiliations:** a Department of Anorectal Surgery, The Third Affiliated Hospital of Zhejiang Chinese Medical University, Hangzhou, China; b Department of General Practice, The First Affiliated Hospital of Zhejiang Chinese Medical University (Zhejiang Provincial Hospital of Traditional Chinese Medicine), Hangzhou, China.

**Keywords:** adult intussusception, colonic submucosal tumor, intestinal lipoma, intestinal obstruction, sigmoid intussusception

## Abstract

**Rationale:**

Intussusception secondary to sigmoid colon lipoma is a rare cause of adult intestinal obstruction. Due to its low prevalence and nonspecific clinical features, diagnosis is often delayed. Early recognition is critical to prevent disease complications and improve patient outcomes.

**Patient concerns:**

We reported a 71-year-old female patient who presented with severe abdominal pain and bloody stool.

**Diagnoses:**

The abdominal computed tomography scan, colonoscopy, and pathological examination indicated that it was a lipoma.

**Interventions:**

The patient underwent sigmoid colectomy with end-to-end coloanal anastomosis.

**Outcomes:**

The patient experienced an uneventful postoperative recovery.

**Lessons:**

In adults, especially the elderly, intussusception secondary to lipoma is rare, and diagnosis is often complicated by intermittent symptoms. Multimodal evaluation (computed tomography, colonoscopy, and histopathology) is essential for diagnostic accuracy. Timely surgical intervention alleviates intestinal obstruction while establishing a definitive diagnosis, necessitating heightened clinical vigilance for this rare entity.

## 1. Introduction

Intussusception is a leading cause of intestinal obstruction in pediatric populations, whereas in adults, especially the elderly, it is rare. Unlike pediatric intussusception, which is typically idiopathic, adult cases are predominantly secondary, with malignancy constituting the majority of etiologies. Significantly, benign neoplasms such as lipomas are exceedingly uncommon as the underlying etiology of intussusception. This report details a rare case of sigmoid intussusception leading to intestinal obstruction in an elderly female, which was diagnosed by CT and colonoscopy, pathologically confirmed to stem from a lipoma, and successfully managed surgically. Therefore, it is crucial to identify these rare causes early through abdominal CT and colonoscopy to prevent disease progression and complications such as intestinal obstruction.

## 2. Case presentation

### 2.1. Clinical manifestations and physical examination

A 71-year-old woman presented to the outpatient clinic with a 3-day history of lower abdominal pain and hematochezia. The patient presented with intermittent, cramping abdominal pain localized to the hypogastric region, accompanied by increased bowel frequency (4–5 daily movements) and scant hematochezia. She reported no nausea, vomiting, or neurological symptoms, with no history of abdominal surgery or malignancy. On physical examination, the patient exhibited lower abdominal tenderness with suspected rebound tenderness, while abdominal muscle rigidity was absent. Rectal examination showed no masses, and the glove was free of blood on withdrawal.

### 2.2. Laboratory and imaging examinations

**Figure 1.**
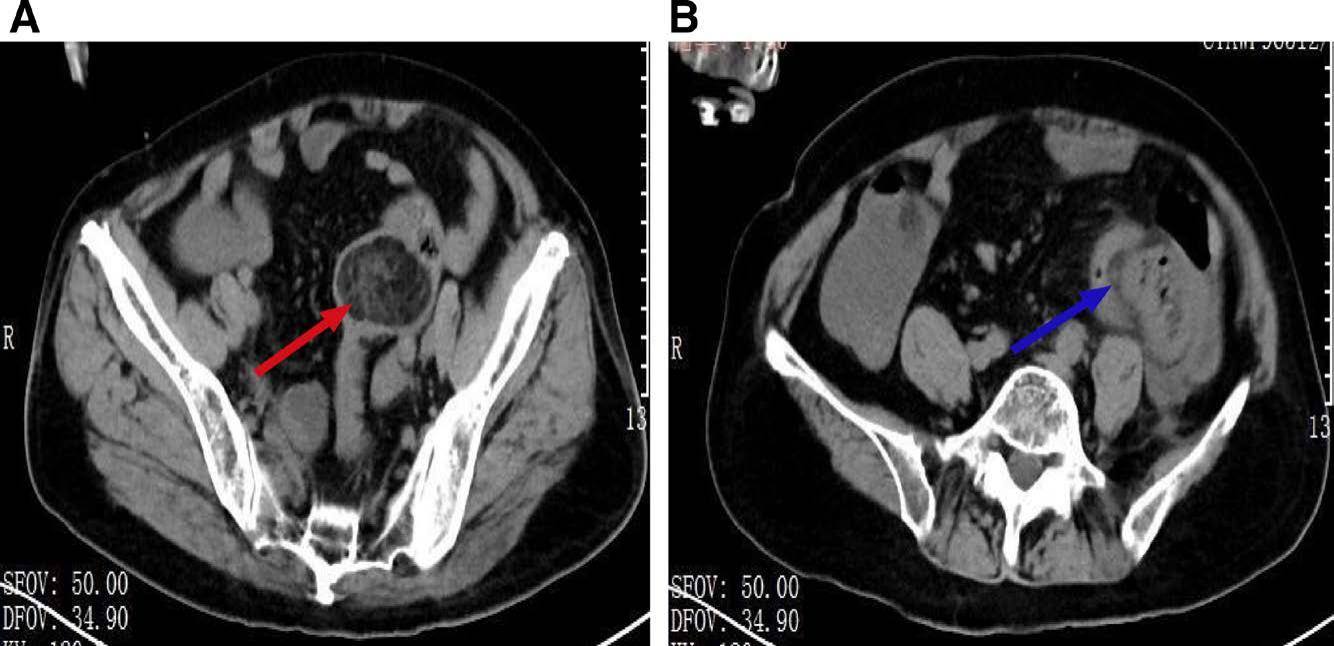
CT examination results. (A) Axial abdominal CT image demonstrates a heterogeneous mass with fatty density within the sigmoid colon (red arrow) and (B) axial abdominal CT image shows intussusception within the abdominal cavity (blue arrow). CT = computed tomography.

Subsequently, the patient underwent laboratory and imaging examinations. Laboratory investigations demonstrated an increase in C-reactive protein level, while the white blood cell count, the percentage of neutrophil, and hemoglobin levels remained within the normal range. Abdominal CT revealed an obstructive, fat-density mass-like lesion (approx. 33.4 mm × 49.9 mm × 32.7 mm) in the sigmoid colon (Fig. 1A), which was associated with local bowel distortion, herniation of adjacent structures, and significant wall edema (Fig. 1B), consistent with a space-occupying lesion causing intestinal obstruction.

### 2.3. Diagnostic and surgical procedure

**Figure 2.**
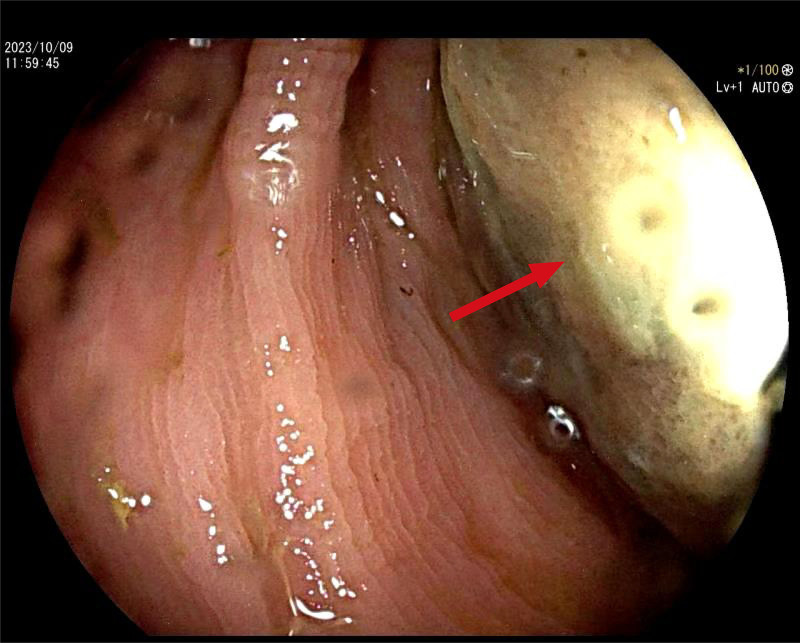
Colonoscopy findings: 35 cm away from the anus, the intestinal wall is congested and edematous, with a covering of yellow secretions (red arrow).

**Figure 3.**
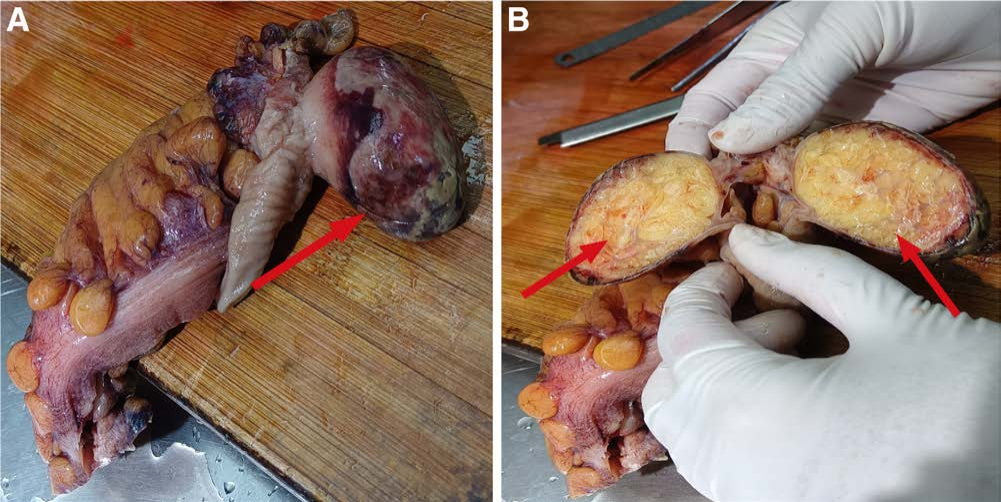
Surgical specimen. (A) Surgical specimen of intestinal lipoma (red arrow) and (B) surgical specimen of intestinal lipoma (cross section; red arrow).

Following the imaging examination, the patient underwent colonoscopy. The procedure revealed intussusception located 35 cm from the anal margin. The affected mucosa appeared congested, dusky, and edematous, with yellow exudate covering the surface, which prevented further endoscopic advancement (Fig. 2). To relieve the obstruction, the patient subsequently underwent sigmoid colectomy with end-to-end coloanal anastomosis (Fig. 3).

### 2.4. Pathology findings

**Figure 4.**
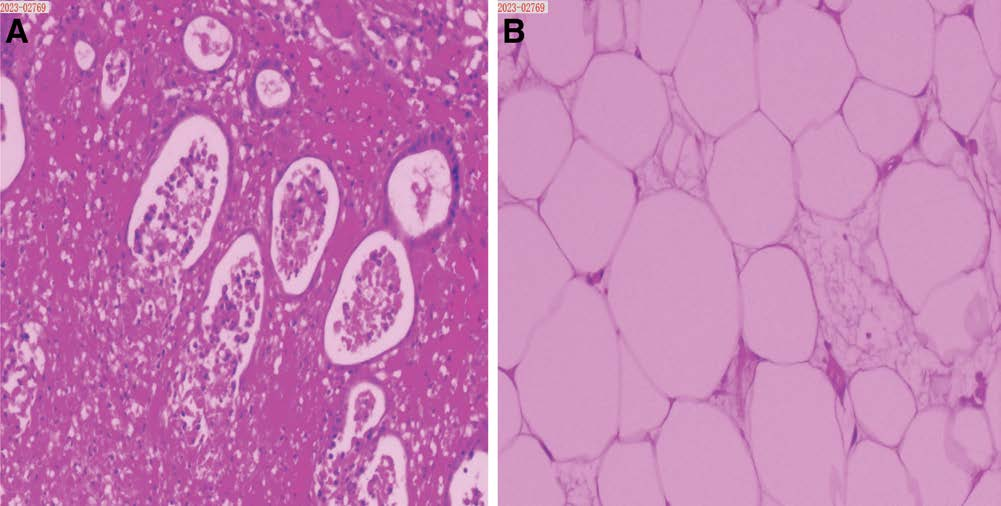
Pathological results. (A) Intestinal tissue pathological section (HE staining, 10×) and (B) pathological section of intestinal lipomatosis (HE staining, 10×). HE = hematoxylin-eosin.

Postoperative histopathological examination revealed a 5 cm × 3 cm fatty nodular lesion within the intestine, consistent with sigmoid colon intussusception secondary to focal lipomatous hyperplasia (Fig. 4). Four peristomal lymph nodes showed chronic inflammatory changes, with no evidence of malignancy.

### 2.5. Patient outcome

Finally, the patient recovered uneventfully without surgical complications and was discharged after clinical stabilization.

## 3. Discussion

Adult intussusception is a rare clinical entity, constituting <5% of all intussusception cases^[1]^ and only 1% to 5% of intestinal obstruction causes in adults.^[2]^ Adult intussusception is predominantly secondary to underlying organic pathologies, with malignant tumors serving as the primary cause, while benign tumors account for fewer cases. Etiologically, intussusception is classified as benign, malignant, and or idiopathic, anatomically, it is categorized into entero-enteric, colo-colonic, ileo-colonic, and ileocecal variants.^[3]^ Among these, the ileocecal junction is the most common anatomical site. Previous cases of intestinal obstruction caused by lipoma were mostly concentrated in the ileocecal region and the right half of the colon,^[4–7]^ while this case occurred in the sigmoid colon. Such a situation is relatively rare in clinical practice.^[8]^ From an anatomical perspective, the lumen of the sigmoid colon is narrow and its peristalsis is strong. The larger pedunculated lipoma acts like a “guiding head,” easily being wrapped by the peristaltic intestinal wall and dragged into the distal lumen, resulting in colonic-colonic type entrapment.

Sigmoid colon lipomas are slow-growing benign neoplasms that typically remain asymptomatic in their early stage.^[9]^ It is important to note that once a tumor grows beyond 2 cm in diameter, nonspecific symptoms like abdominal pain, gastrointestinal bleeding, or intussusception may arise.^[10,11]^ These symptoms show significant individual differences. Some patients may only experience intermittent lower abdominal pain, which might be mistaken for common conditions such as intestinal motility disorders or spasms.^[12]^ Conversely, others present with signs of incomplete intestinal obstruction, such as nausea, vomiting, and cessation of flatus and bowel movements, overlapping with clinical features of colorectal malignancies or adhesions.^[13]^ When the tumor diameter exceeds 4 cm, pedunculated colorectal lipomas, in particular, may slide within the intestinal lumen and readily lead to intussusception or intestinal obstruction. In the present case, the intestinal lipoma remained asymptomatic until it reached approximately 5 cm in size and triggered intussusception, manifesting as abdominal pain and hematochezia. Although the narrower lumen of the left colon theoretically predisposes it to a higher risk of obstruction, the insidious and asymptomatic course in this case posed a significant challenge to early diagnosis.

Previous reports have documented cases where patients initially presented with intermittent abdominal pain, leading to misdiagnosis as functional dyspepsia, definitive diagnosis was achieved only after extended observation and advanced diagnostic evaluations.^[14]^ These cases highlight the insidious presentation and diagnostic complexity of sigmoid colon lipomas, emphasizing the significant challenge in differentiating lipoma-induced intussusception from other conditions in adults. Both lipomas and malignant tumors of the sigmoid colon may present with abdominal pain, hematochezia, or intraluminal masses.^[15]^ However, malignant neoplasms typically demonstrate rapid progression and systemic symptoms (e.g., fatigue, anemia, and weight loss), contrasting sharply with lipomas’ indolent growth pattern. Clinical symptoms and initial assessments seldom permit reliable differentiation between these entities. Computed tomography (CT) is required to identify characteristic lipoma features – well-defined, homogeneous fat-density lesions within the colonic lumen showing no contrast enhancement. However, when complicated by intussusception, intestinal edema, or exudation, lipomas’ characteristic fat-density signature may become obscured, potentially reducing diagnostic accuracy.^[16]^

Based on this case, we recommend that colonoscopy be performed as early as possible in patients presenting with symptoms such as abdominal pain or bloody stool, once contraindications have been ruled out. Additionally, even in the absence of symptoms, it is advisable for individuals over 40 years of age to undergo colonoscopy every 5 years. Such a strategy facilitates early detection of the disease. If lesions are small in diameter, they can be resected during colonoscopy, thereby avoiding the need for intestinal resection.

Colonoscopy facilitates lesion localization and characterization. However, its diagnostic value is frequently limited by inadequate bowel preparation in patients with severe abdominal pain. The predominantly submucosal growth of lipomas also limits endoscopic biopsy adequacy.^[9]^ Most critically, surface congestion, erosion, or ulceration of lipomas obscure diagnostic features, increasing the risk of misdiagnosis as sigmoid adenocarcinoma.^[12,13]^ In cases where noninvasive diagnostics yield inconclusive results, surgical intervention becomes essential for definitive diagnosis and treatment. Intraoperative assessment of tumor morphology and texture combined with rapid frozen section analysis enables preliminary characterization. However, final diagnosis requires postoperative histopathological and immunohistochemical evaluations. Pathological examination typically demonstrates mature adipocytes, confirming differentiation from malignancies and informing therapeutic planning.

## 4. Conclusion

Adult intestinal obstruction caused by sigmoid colon lipomas is frequently misdiagnosed owing to its rarity and nonspecific symptoms such as abdominal pain and hematochezia, which mimic colorectal malignancies. Both CT and colonoscopy face diagnostic limitations: inflammatory changes may obscure CT findings, while endoscopic biopsies often yield insufficient tissue for definitive diagnosis. Heightened clinical suspicion is paramount, requiring integration of patient history, physical examination, and imaging findings. When noninvasive modalities prove inconclusive, prompt surgical intervention is warranted. This approach simultaneously alleviates obstruction and enables definitive histopathological diagnosis, ultimately improving patient outcomes.

## Acknowledgments

We acknowledge AI service of DeepSeek for providing us with language polishing.

## Author contributions

**Writing – original draft:** Chenchen Zhang.

**Writing – review & editing:** Xiaojuan Tong, Dong Zhai.
